# *Fusarium sacchari* CFEM Proteins Suppress Host Immunity and Differentially Contribute to Virulence

**DOI:** 10.3390/ijms252312805

**Published:** 2024-11-28

**Authors:** Tianshu Hong, Shichao Wang, Zhiyuan Luo, Qianqian Ren, Deng Wu, Lulu Wang, Yixue Bao, Wei Yao, Muqing Zhang, Qin Hu

**Affiliations:** 1 State Key Laboratory for Conservation and Utilization of Subtropical Agro-bioresources, Guangxi University, Nanning 530004, China; 2 Guangxi Key Laboratory of Sugarcane Biology, Nanning 530004, China; 3 College of Agronomy, Guangxi University, Nanning 530004, China

**Keywords:** *Fusarium sacchari*, sugarcane, CFEM, virulence differentiation, host immunity

## Abstract

The pathogen *Fusarium sacchari* is responsible for the devastating pokkah boeng disease, which causes significant economic losses in sugarcane production. However, the mechanisms by which it affects plant immunity remain largely unknown. Common in Fungal Extracellular Membrane (CFEM) domain proteins have been implicated in fungal growth, infection processes, and pathogenicity. In this study, we identified three FsCFEM proteins (Fs08184, Fs10706, and Fs13617) that mediate the broad-spectrum suppression of the immune responses induced by typical effectors. A further analysis demonstrated that Fs08184, Fs10706, and Fs13617 suppressed host immunity through two potential iron-binding sites conserved in CFEM family members, characterized by Asp and Phe residues in Fs08184, Fs10706, and Fs13617. Additionally, the Asp and Phe residues within the iron-chelating site were necessary for the iron acquisition of *F. sacchari* and contributed to creating low-free-iron conditions at the interface of plant and pathogen interactions. It appeared that *F. sacchari* might employ Asp-Phe-type CFEM members to influence host iron homeostasis to suppress host immunity and to facilitate its successful colonization.

## 1. Introduction

Pokkah boeng disease (PBD) is the most common fungal disease of sugarcane, mainly caused by *Fusarium sacchari.* Since the discovery of the disease in Indonesia in 1896, PBD has been widely distributed around the world, seriously threatening the sugar industry. In China, the incidence rate of PBD is as high as 95%, causing a 30–48% reduction in sugarcane yield and a two to four percent drop in sugar content [1]. Initially, sugarcane leaves infected with PBD exhibit yellowing of the heart leaves. In severe cases, necrosis may occur at the tips of sugarcane plants, potentially leading to the wilting and death of the entire plant.

Plants have a specialized defense system, and pathogenic microorganisms have continuously produced new effectors in the co-evolution arms race with plants to break through the plant immune system. When pathogens invade plants, effectors are transferred to plant cells to suppress the host immune responses or manipulate host cell physiology to facilitate invasion and colonization. Although effectors are key weapons in suppressing PAMP-triggered immunity (PTI), they can also be recognized by plant resistance proteins (R proteins), triggering a second layer of defense response termed effector-triggered immunity (ETI). Thus, the ability of a pathogen to successfully invade the host depends on the special effectors that can escape the recognition of the host R protein.

With the advancement of whole-genome sequencing technology, numerous effectors have been identified in plant pathogens. Fungal effector proteins exhibit various conserved motifs, including crinkler (CRN), lysin (LysM), necrosis-inducing Phytophthora protein (NPP), and Common in Fungal Extracellular Membrane (CFEM) domains. CFEM proteins are fungal-specific and consist of 60 amino acids, incorporating eight cysteine residues with a consensus sequence of PxC [A/G] x_2_Cx_8-12_Cx_1-3_[x/T] Dx_2-5_CxCx_9-14_Cx_3-4_Cx_15-16_ [2]. Numerous studies have demonstrated that CFEM is closely linked to fungal pathogenicity. For instance, the MoCDIPs of *Magnaporthe oryzae* are capable of inducing host cell death [3]. Both MoPth11 and its homolog, *MoPth11-like,* facilitate adherent cell formation, while the structural domain of Pth11 containing CFEM is crucial for *Magnaporthe oryzae*’s virulence [4]. The deletion of *BcCFEM1* in *Botrytis cinerea* enhances host resistance against this pathogen, as this protein induces programmed cell death in tobacco leaves through this mechanism [5]. Members of the *Verticillium dahliae* CFEM family, specifically *VdSCP76* and *VdSCP77*, inhibit necrosis induced by other *Verticillium dahliae* effector proteins or Bcl-2-associated X protein (BAX) in tobacco cells [6]. FgCFEM1 secreted by *Fusarium graminearum* enhances pathogenicity by binding to the ZmWAK17 disease resistance gene in maize, thereby compromising its disease resistance function [7]. In addition, CgCyw14, which has a CFEM structure in *Candida glabrata*, is crucial for intracellular iron maintenance, adhesion capacity, and overall pathogenicity [8]. Six proteins containing the CFEM structural domain have been identified in *Candida albicans*. Through the construction of knockout mutants, researchers discovered that these mutants exhibited significant defects in biofilm structure and a diminished ability to adhere to both inert and biological surfaces. The capacity to form mycelia is regarded as an important characteristic of *Candida glabrata* virulence; thus, the genes encoding these CFEM protein families that are up-regulated during mycelial expression are likely to play a vital role in the virulence of this organism [9,10]. The CFEM structural domain bears a resemblance to human G protein-coupled receptor-like (GPR) epidermal growth factor (EGF), functioning either as an extracellular receptor or signaling factor or as an adhesion molecule in host–pathogen interactions [2,4,11]. Thus, the proteins containing the CFEM domain play crucial roles in pathogenic fungi. However, research on the effectors of the CFEM domain in *Fusarium sacchari* remains quite limited at present.

Like the mechanisms identified in other phytopathogens, *F. sacchari* secretes numerous effectors to manipulate host immunity during the course of infection. Data mining of the whole genome of *F. sacchari* has revealed that the *F. sacchari* strain CNO-1 encodes 316 candidate secreted effector proteins (CSEPs, proteins with 50–300 amino acid residues and an N-terminal signal peptide, lacking transmembrane domains and glycosyl-phosphatidyl-inositol (GPI) anchor sites). Notably, some of these CSEPs have been demonstrated to function as effectors that manipulate host immunity [12]. For instance, the protein Fs03538 is targeted in the host nucleus, suppressing Bcl-2-associated X protein (BAX)-induced cell death and defense responses in *Nicotiana benthamiana* [13]. *FsPL* encodes a pectate lyase that is secreted by *F. sacchari*. A further analysis demonstrates that *FsPL* is an important virulence factor of *F. sacchari* and could trigger the typical PTI response and cell necrosis in *N. benthamiana* in an SOBIR1-BAK1-dependent manner [14]. Fs00367 is a *Fusarium* genus-specific effector that plays a crucial role in the full virulence of *F. sacchari* and has the ability to suppress host immunity responses by directly interacting with and inhibiting ScPi21-induced cell death [15]. Twenty CFEM domain-containing proteins have been identified from *F. sacchari*, and these members exhibit significant virulence differentiation and pathogenicity in sugarcane [16]. In this study, we aimed to elucidate the mechanisms underlying the virulence differentiation of CFEM members in *F. sacchari*. We identified two potential iron-binding sites that are conserved among these CFEM family members, which mediate broad-spectrum cell death suppression. These sites, characterized by aspartic acid (Asp) and phenylalanine (Phe) residues, were found to be closely associated with the virulence differentiation of CFEM members in *F. sacchari*. Our findings will further contribute to understanding the molecular mechanisms involved in plant–pathogen interactions while highlighting the significance of CFEM proteins.

## 2. Results

### 2.1. CFEM Family Members Fs08184, Fs10706, and Fs13617 Display Broad-Spectrum Cell Death Suppression

**Figure 1:**
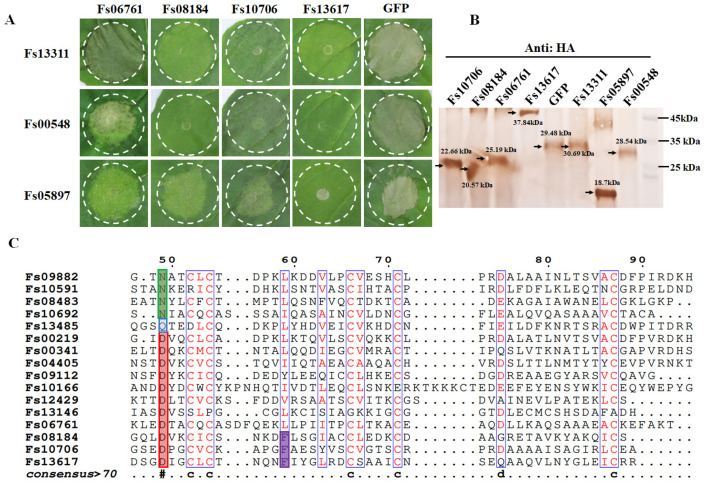
**Identification of the broad-spectrum cell death suppression of CFEM protein family members in *F. sacchari.*** (**A**) Suppression activity of Fs06761, Fs08184, Fs110706, and Fs13617 against known *F. sacchari* cell-death-inducing proteins including Fs13311, Fs00548, and Fs05897 in *N. benthamiana* leaves. Transiently expression of GFP was used as negative control. White circles outline the infiltrated area. (**B**) The efficiency of transient expression of the specified proteins in *N. benthamiana* leaves was confirmed through Western blot analysis using an HA antibody. (**C**) Sequence alignment of the FsCFEMs from *F. sacchari* reveals that aspartic acid (Asp) and phenylalanine (Phe) residues within the CFEM domain are both specific and conserved across the three members of Fs08184, Fs10706, and Fs13617.

Based on the *Fusarium sacchari* genome sequence [16], 316 candidate secreted effector proteins were identified that contained an N-terminal signal peptide but lacked the transmembrane domain and glycosylphosphatidylinositol (GPI) anchor sites. In total, 20 effectors that contained the conserved CFEM domain were identified and 16 *FsCFEMs* were cloned, including *F. sacchari* CFEM effectors Fs06761, Fs08184, Fs10706, and Fs13617, which were identified as effector proteins that could suppress the programmed cell death (PCD) triggered by BCL2-associated X protein (BAX) in *Nicotiana benthamiana*. Moreover, 15 CFEM members failed to fully suppress cell death caused by BAX. To further examine the cell-death-inhibiting functions of the four CFEM-containing effectors (Fs06761, Fs08184, Fs10706, and Fs13617), each was transiently co-expressed with three *F. sacchari* cell-death-inducing proteins (Fs13311, Fs00548, and Fs05897) in *N. benthamiana* by agroinfiltration, respectively. As shown in Figure 1A, Fs08184, Fs10706, and Fs13617 showed the broadest spectrum of cell death suppression. An immunoblotting analysis confirmed the effective translation of all the tested effectors in *N. benthamiana* (Figure 1B).

### 2.2. Fs08184, Fs10706, and F13617 Inhibit Host Immunity Through Conserved Iron-Binding Sites in the CFEM Family

Previous studies have shown that the CFEM effectors of *Candida albicans* are essential for capturing heme iron from host proteins and transferring it through the cell wall to the cell membrane. The functionality of these CFEM effectors is contingent upon a conserved aspartic acid (Asp, D) residue within the CFEM domain [17]. In contrast, findings related to *Verticillium dahlia* indicate that it is primarily the asparagine residues (Asn, N) in the CFEM domain that facilitate this function, and *V. dahliae* might employ N-type CFEM members to suppress host immunity and to promote successful colonization [6]. These studies collectively illustrate that the mechanisms underlying virulence differentiation among proteins in the CFEM family differ across various pathogens.

The existing results showed that twenty CFEM effectors of *F. sacchari* exhibited different inhibitory effects on cell death induced by different effectors. Notably, Fs08184, Fs10706, and F13617 displayed the broadest-spectrum cell death suppression compared to other members. Therefore, the virulence of these FsCFEM effectors is likely to be different, and Fs08184, Fs10706, and F13617 are the three CFEM members that play crucial roles in host immunity manipulation via their broad-spectrum ability to suppress cell death. To further investigate the underlying mechanism(s) conferring the broad-spectrum cell death suppression ability of Fs08184, Fs10706, and F13617, amino acid sequence alignment was performed to identify the possible sites associated with differences in virulence. As shown in Figure 1C, the residues diverged to D and Phe (F) sites in Fs08184, Fs10706, and F13617, indicating that both Asp and Phe might contribute significantly to the broadest-spectrum host immunity suppression ability of Fs08184, Fs10706, and F13617 relative to the other CFEM members of *F. sacchari*.

**Figure 2:**
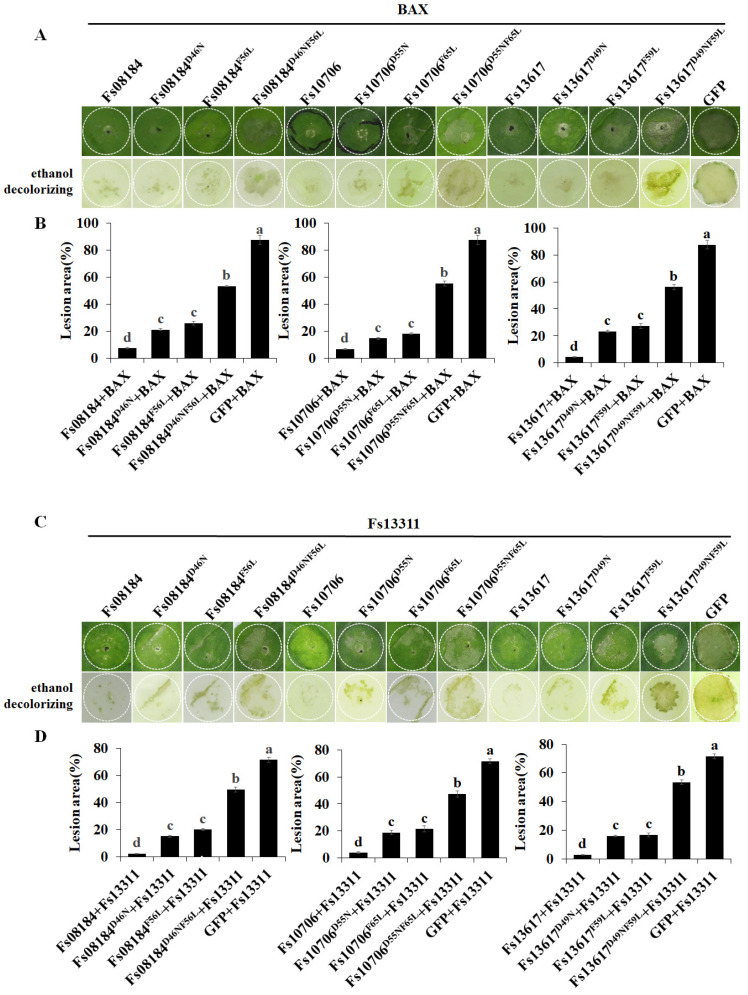
**Functional analyses of two potential iron-binding sites Asp and Phe in cell death suppression.** (**A**) Cell death suppression activity of native and Asn > Asp, Phe > Leu site-directed mutant proteins of Fs08184, Fs10706, and Fs13617 was detected by co-expressing them transiently with BAX in 4-week-old *N. benthamiana* leaves. GFP and BAX were used as controls. White circles outline the infiltrated area. (**B**) The cell death areas of the indicated infiltrated regions were calculated at 5 d post inoculation and are expressed as the ratio of the lesion area to the infiltrated area. Values are the means ± SD; n = 9. (**C**) Cell death suppression activity of native and Asn > Asp, Phe > Leu site-directed mutant proteins of Fs08184, Fs10706, and Fs13617 were detected by co-expressing them transiently with Fs13311 in 4-week-old *N. benthamiana* leaves. GFP and Fs13311 were used as controls. White circles outline the infiltrated area. (**D**) The cell death areas of the indicated infiltrated regions were calculated at 5 d post inoculation and are expressed as the ratio of the lesion area to the infiltrated area. Values are the means ± SD; n = 9. Different letters indicate statistically significant differences among samples (one-way ANOVA, *p* < 0.05).

To further investigate the relationship between the conserved sites and suppression of immunity, the mutated sequences of *Fs08184^D46N^* (Asp46 substituted to Asn), *Fs08184^F56L^* (Phe56 substituted to Leu), *Fs08184^D46NF56L^* (Asp46 and Phe56 substituted to Asn and Leu, respectively), *Fs10706^D55N^* (Asp56 substituted to Asn), *Fs10706^F66L^* (Phe66 substituted to Leu), *Fs10706^D55NF65L^* (Asp56 and Phe66 substituted to Asn and Leu, respectively), *Fs13617^D49N^* (Asp49 substituted to Asn), *Fs13617^F59L^* (Phe66 substituted to Leu), and *Fs13617^D49NF59L^* (Asp49 and Phe59 substituted to Asn and Leu, respectively) were generated and co-expressed with BAX and Fs13311 in *N. benthamiana* leaves. The results showed that only the two-site mutant proteins Fs08184^D46NF56L^, Fs10706^D56NF66L^, and Fs13617^D49NF59L^ completely lost the ability to suppress cell death induced by BAX (Figure 2A,B) and Fs13311 (Figure 2C,D) in *N. benthamiana* leaves, and the single-site mutant proteins Fs08184^D46N^, Fs08184^F56L^, Fs10706^D56N^, Fs10706^F66L^, Fs13617^D49N^, and Fs13617^F59L^ only partially lost their ability to inhibit BAX and Fs13311-induced cell death (Figure 2).

**Figure 3:**
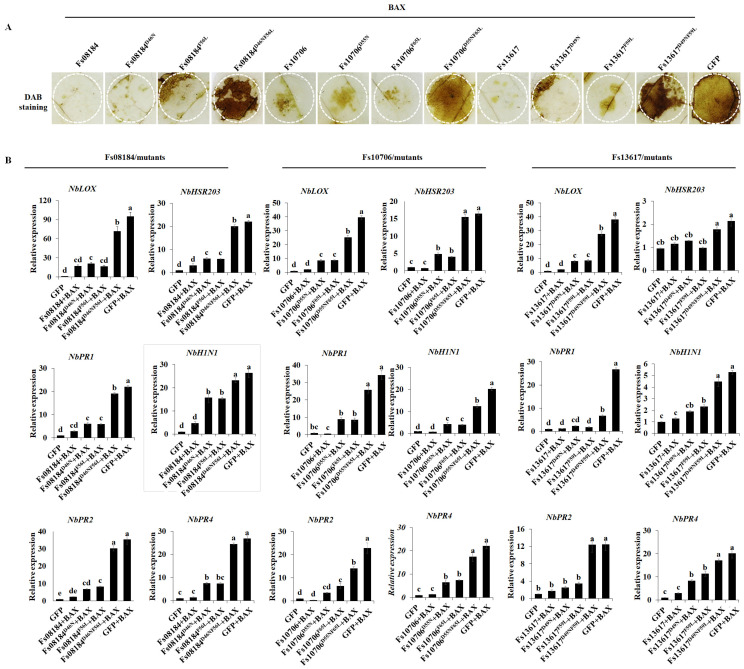
**The inhibition of host immunity by Fs08184, Fs10706, and F13617 requires the conserved iron-binding sites Asp and Phe.** (**A**) ROS accumulation after transient co-expression of native and Asn > Asp, Phe > Leu site-directed mutant proteins of Fs08184 and Fs10706 and with BAX in 4-week-old *N. benthamiana* leaves was determined by 3,3′-diaminobenzidine (DAB) staining. GFP and BAX were used as controls. (**B**) The expression levels of defense-related marker genes including *NbLOX*, *NbHSR203*, *NbHIN1*, *NbPR1*, *NbPR2,* and *NbPR4* in *N. benthamiana* leaves transiently expressing the indicated constructs at 2 days post inoculation. *NbEFα* was used as the endogenous control. Values are the means ± SD; n = 3. Different letters indicate statistically significant differences among samples (one-way ANOVA, *p* < 0.05).

As expected, the host immunity responses represented by reactive oxygen species (ROS) burst (Figure 3A) and the up-regulation of defense-related genes including *NbLOX*, *NbHSR203*, *NbHIN1*, *NbPR1*, *NbPR2,* and *NbPR4* (Figure 3B) were most significantly restored in the *N. benthamiana* leaves co-expressing the two-site mutant proteins (Fs08184^D46NF56L^, Fs10706^D56NF66L^, or Fs13617^D49NF59L^) with BAX, followed by the *N. benthamiana* leaves co-expressing the single-site mutant proteins, and lastly the *N. benthamiana* leaves co-expressing the native proteins with BAX (Figure 3). The above results strongly suggested that the potential iron-binding sites Asp and Phe in the CFEM domain are related to the function of suppressing host immunity and contributed to the virulence differentiation of the CFEM effector family in *F. sacchari*.

### 2.3. The Diverged Iron-Binding Sites in Fs08184, Fs10706, and Fs13617 Also Contributed to the Virulence of F. sacchari in Sugarcane

**Figure 4:**
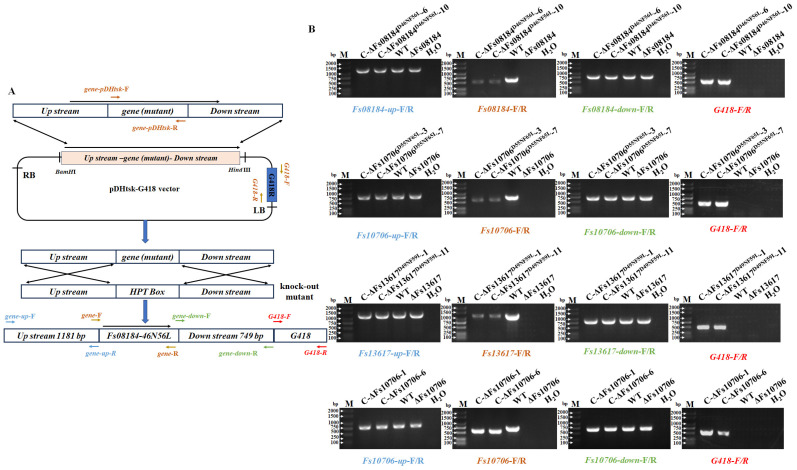
***A. tumefaciens*-mediated transformation of *F. sacchari*.** (**A**) The schematic diagram illustrates the strategy employed to generate complementary mutants in the knockout mutant strain. (**B**) PCR identification of the positive complementary mutants using the primer pairs indicated in (**A**).

**Figure 5:**
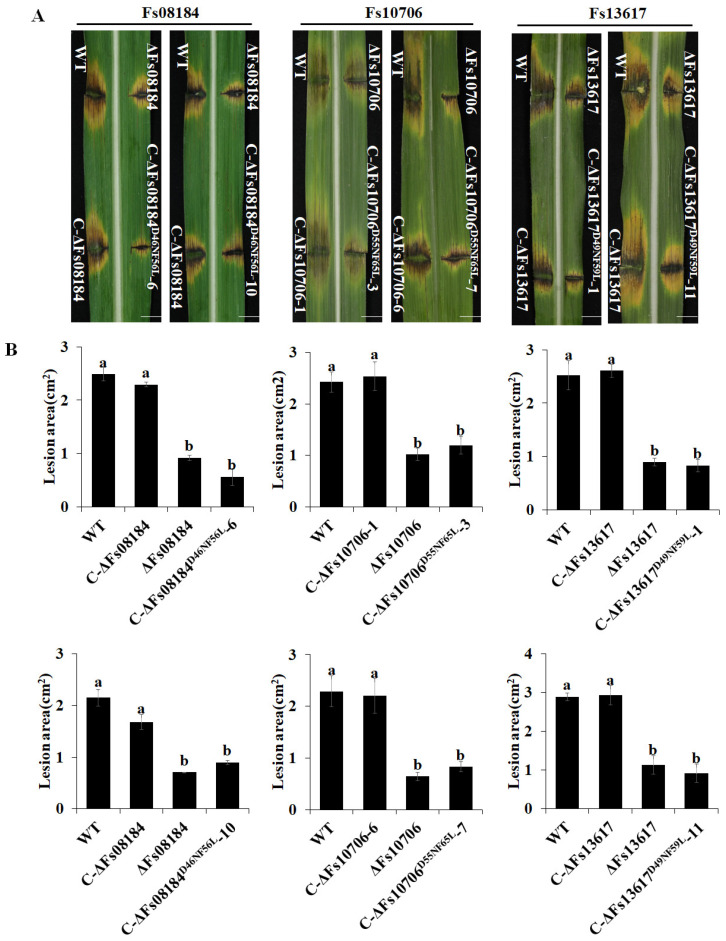
**Asp and Phe residues in CFEM domain of Fs08184, Fs10706, and Fs13617 are critical for the virulence of *F. sacchari.*** (**A**) Pathogenicity assays of the knockout, native complementary, and Asn > Asp, Phe > Leu site-directed complementary mutants of *Fs08184*, *Fs10706,* and *Fs13617.* Disease symptoms of the wild-type strain CNO-1 (WT), as well as ΔFsCFEM and FsCFEM complementary mutants. Representative photographs were taken at 7 days post inoculation. Bar: 1 cm. (**B**) Statistical analysis of the lesion areas caused by the indicated *F. sacchari* strains at 7 days post inoculation. Values are the means ± SD; n = 6. Different letters indicate statistically significant differences among samples (one-way ANOVA, *p* < 0.05).

Our previous study demonstrated that Fs08184, Fs10706, and Fs13617 are the virulence factors needed for the full virulence of *F. sacchari* in sugarcane, which was evidenced by the significantly weakened lesion areas caused by *Fs08184*, *Fs10706,* and *Fs13617* knockout strains of *F. sacchari* [16]. Therefore, the complemented strains C-ΔFs08184^D46NF56L^, C-ΔFs10706^D56NF66L^, and C-ΔFs13617^D49NF59L^, in which Asp and Phe were substituted to Asn and Leu, respectively, were generated to explore the role of these two divergent sites in the virulence of *F. sacchari* in sugarcane (Figure 4A). The positive complemented mutant strains were verified by PCR using the corresponding primer pairs as indicated in Figure 4A,B, and an in vitro inoculation experiment was carried out in sugarcane leaves to evaluate the pathogenicity of different mutants. As indicated in Figure 5A, the lesion areas of the sugarcane leaves inoculated with the complemented strains C-ΔFs08184, C-ΔFs10706, and C-ΔFs13617 were similar to that of the wild-type strain, but were the smallest in those of C-ΔFs08184^D46NF56L^, C-ΔFs10706^D56NF66L^, and C-ΔFs13617^D49NF59L^, indicating that the strains with the mutation of the potential iron-binding site (D>>N and F>>L) failed to increase virulence in the sugarcane. Statistical analyses of the lesion area also supported this conclusion (Figure 5B). These results demonstrated that the conserved sites Asp and Phe of FsCFEMs are necessary for *F. sacchari* to exert pathogenicity.

### 2.4. F. sacchari May Employ Asp-Phe-Type CFEM Members to Influence Iron Capture and Iron Homeostasis in the Interaction Between Pathogen and Host

**Figure 6:**
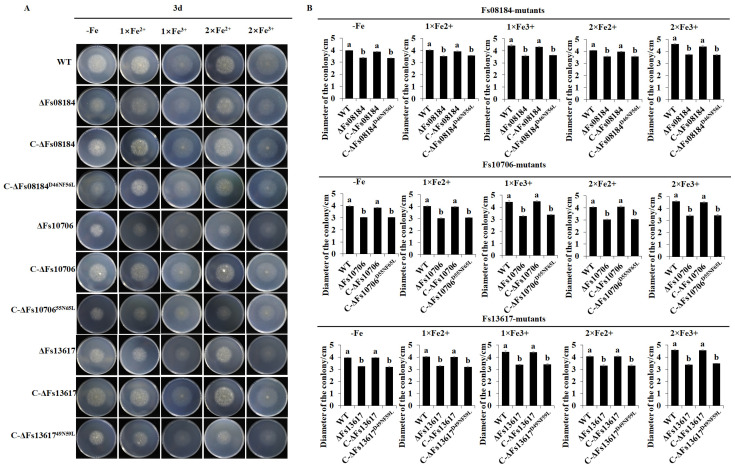
**Asp-Phe-type CFEM members are involved in iron acquisition and utilization in *F. sacchari.*** (**A**) The growth of the knockout, native complementary, and Asn > Asp, Phe > Leu site-directed complementary mutants of *Fs08184*, *Fs10706,* and *Fs13617*on Czapek-DOX medium containing different concentrations of Fe^2+^ and Fe^3+^ for 3 days. (**B**) Statistical analysis of the vegetative growth of the indicated mutants that were cultured on Czapek-DOX medium containing different concentrations of Fe^2+^ and Fe^3+^ for 3 days. Values are the means ± SD; n = 3. Different letters indicate statistically significant differences among samples (one-way ANOVA, *p* < 0.05).

**Figure 7:**
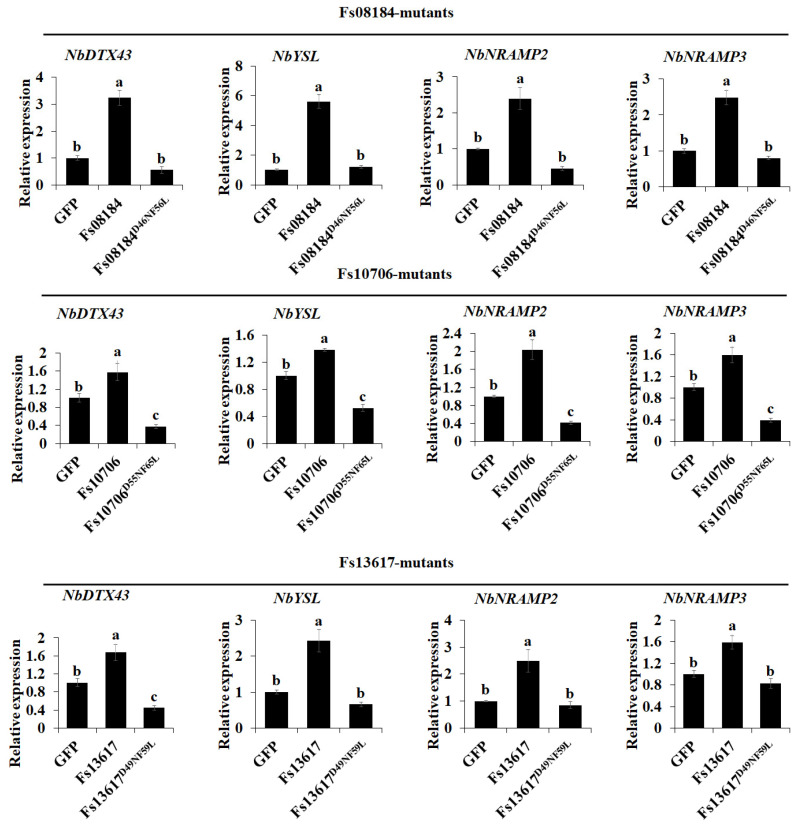
**Asp-Phe-type CFEM members are also involved in iron homeostasis in *N. benthamiana*.** The transcription levels of genes associated with iron transport in *N. benthamiana* leaves, following the transient expression of the indicated proteins for two days, were assessed using RT-qPCR. Values are the means ± SD; n = 3. Different letters indicate statistically significant differences among samples (one-way ANOVA, *p* < 0.05).

Iron homeostasis plays a key role in plant immunity during the competition between pathogens and plants [18]. Some fungal CFEM proteins, for instance, Rbt5, Rbt51, Pga7, and Cas1 from *Candida albicans* [19], CFEM2, CFEM3, and CFEM6 from *Candida parapsilosis* [20], and VdSCP76 and VdSCP77 from *V. dahliae* [6], are reported to be involved in iron acquisition or iron homeostasis in pathogen–host interaction to facilitate its colonization. We therefore further investigated the potential roles of the two conserved sites Asp and Phe in iron capture and iron homeostasis and their relationship with cell death suppression and virulence differentiation in Fs08184, Fs10706, and Fs13617. Firstly, the knockout and complementary mutant strains were cultured under iron-deficient and iron-sufficient conditions. At 3 days after inoculation, as shown in Figure 6A, regardless of the iron-deficient, normal iron, or iron-sufficient conditions or the presence of Fe^2+^ or Fe^3+^, the colony sizes of the knockout mutants *ΔFs08184*, *ΔFs10706,* and *ΔFs13617* were always smaller than that of the wild-type strain, while the colony sizes of the complemented strains C-*ΔFs08184*, C-*ΔFs10706*, and C-*ΔFs13617* recovered to the same level as the wild-type strain. However, the colony sizes of the two-site mutant type of complemented mutants C-*ΔFs08184^D46NF56L^*, C-*Δ*Fs10706^D56NF66L^, and C-*Δ*Fs13617^D49NF59L^ did not fully recover to the wild-type level, and their growth was still smaller than that of the wild-type strain. The statistical analyses of the colony sizes also supported this conclusion (Figure 6B). These results suggested that the Asp and Phe residues are essential for the utilization of iron in *F. sacchari*. Subsequently, we examined the expression of iron homeostasis-related genes including *NbDTX43*, *NbYSL1*, *NbNramp2,* and *NbNramp3* in *N. benthamina* in response to the transiently expressed Fs08184, Fs10706, and Fs13617 and the different mutation types. As expected, the expression levels of *NbDTX43*, *NbYSL1*, *NbNramp2,* and *NbNramp3* were significantly up-regulated at 48 h after the transient expression of Fs08184, Fs10706, and Fs13617 in *N. benthamina* and showed no response to the transient expression of C-ΔFs08184^D46NF56L^, C-ΔFs10706^D56NF66L^, and C-ΔFs13617^D49NF59L^ (Figure 7). Therefore, based on the results, we hypothesized that the Asp and Phe residues in the iron-chelating site were necessary for the iron acquisition of *F. sacchari* and contributed to the creation of low-free-iron conditions at the interface of plant and pathogen interactions; as feedback, genes involved in iron homeostasis were up-regulated in the host, and *F. sacchari* might employ Asp-Phe-type CFEM members to influence host iron homeostasis to suppress host immunity and to facilitate successful colonization. 

## 3. Discussion

Since the inception of plant–pathogen interactions, a fierce but silent war has been waged between pathogens and plants [21]. In this subtle war, effector proteins from pathogens serve as the first instance of communication between plants and pathogens, to some extent, determining the outcome of this war. When the effector proteins are recognized by the NLR proteins of the plant and initiate the immune responses, the pathogens lose the war. Conversely, when the effector proteins successfully evade recognition by the plant’s surveillance system and suppress the host immune responses, the pathogens exert successful colonization and expansion [22]. Consequently, to ensure their survival, pathogens must continuously evolve new effector proteins to counteract the ever-adapting immune systems of plants [23]. Therefore, the identification of disease-related effector proteins in pathogens represents a significant breakthrough in our understanding of the mechanisms underlying plant–disease interactions and enhancing plant resistance.

The CFEM (Common in Fungal Extracellular Membrane) domain comprises approximately 60 amino acids with eight conserved cysteine residues motif (PxC [A/G] x2Cx8-12Cx1-3[x/T] Dx2-5CxCx9-14Cx3-4Cx15-16). This domain is specific to the fungal kingdom and frequently characterized as membrane proteins [24]. In fact, the localization, biofunction, and working mechanisms of CFEM proteins in the fungal community have been expanded in recent years. The primary function of CFEM proteins is reported to be associated with surface sensing and signal transduction, as they are localized at the cell membrane or anchored to the surface of the cell membrane or wall via glycosylphosphatidylinositol (GPI) anchor sites [25]. For instance, a secreted CFEM protein MgACI1 from *Magnaporthe grisea* has been identified as a putative membrane-bound receptor or adhesion molecule participating in cAMP signal transduction and further influencing appressorium differentiation via directly interacting with adenylate cyclase (MAC1) [26]. Another well-studied CFEM protein, *CgCsa* from *Colletotrichum gloeosporioides*, plays a crucial role in the regulation of Fe^3+^ homeostasis and the growth of mycelium. The deletion of *CgCsa* results in significant deficits in hyphal growth, conidial yield, and conidial germination; abnormal appressorium with elongated bud tubes; and the virulence of *C. gloeosporioides*. Providing an excess of Fe^3+^ could partially recover the defective phenotypes of the *CgCsa* knockout mutant, indicating the association between CFEM protein and iron homeostasis in *C. gloeosporioides* [27]. In fact, an increasing number of reports indicate that not all CFEM proteins are exclusively anchored to the fungal cell membrane or cell wall. Some CFEM proteins have also been found to target the host’s apoplast or cytoplasm, thereby influencing the host’s immune responses to facilitate infection. For example, FgCFEM1 from *Fusarium Graminearum* has been proven to be an apoplastic effector in the host apoplast during *F. graminearum*–maize interaction. A further analysis demonstrated that FgCFEM1 could directly interact with two secreted maize proteins, ZmLRR5 and ZmWAK17ET, to compromise ZmWAK17-mediated resistance [7]. Recently, it has been reported that *Colletotrichum fructicola* secreted a CFEM effector, CfEC12, into the host nucleus, and the transient expression of CfCE12 could suppress BAX-induced cell death and ROS burst in *N. benthamiana* leaves, suggesting that CfCE12 could suppress the host defense responses. Further experiments have proven that CfEC12 interacts with apple MdNIMIN2, an NIM1-interacting (NIMIN) protein that modulates NPR1 activity in response to salicylic acid. Notably, CfEC12 disrupts the interaction between MdNIMIN2 and MdNPR1 by competitively binding, thereby interrupting the SA-mediated defense signaling pathway [28]. In this work, we identified three CFEM members, Fs08184, Fs10706, and Fs13617, from the 19 CFEM members from *F. sacchari* that are capable of broadly suppressing the host immune response (Figure 1A) and that have been established as essential for the complete virulence of *F. sacchari* (Figure 5A). The transient expression of these three members in *N. benthamiana* inhibited BAX/Fs13311/Fs00548/Fs05897-triggered cell death, as well as reactive oxygen species (ROS) burst and the expression of defense-related genes, suggesting that Fs08184, Fs10706, and Fs13617 functioned as effectors to suppress host immunity (Figure 2 and Figure 3). Simultaneously, we also observed significant growth inhibition occurred in the knockout mutants of *Fs08184*, *Fs10706,* and *Fs13617* under both iron-deficient and iron-rich conditions, indicating that these three effectors were involved in the utilization of Fe^2+^ or Fe^3+^ of *F. sacchari* (Figure 6). Furthermore, the transient expression of Fs08184, Fs10706, and Fs13617 also resulted in the up-regulation of genes associated with iron homeostasis in *N. benthamiana* (Figure 7). We hypothesized that Fs08184, Fs10706, and Fs13617 were necessary for the iron acquisition of *F. sacchari* and contributed to the creation of low-free-iron conditions at the interface of plant and pathogen interactions; as feedback, genes involved in iron homeostasis were up-regulated in the host, and *F. sacchari* might employ Fs08184, Fs10706, and Fs13617 to influence host iron homeostasis to suppress host immunity and to facilitate successful colonization. In our previous research, we identified that Fs08184, Fs10706, and Fs13617 are localized in both the cell membrane and nucleus, implying that these three CFEM members not only fulfill the role of membrane-localized CFEM proteins but also potentially function as intracellular effector proteins capable of interacting with the host protein(s) to modulate host immunity responses [16]. Therefore, the inhibited defense response and alterations in iron homeostasis induced by Fs08184, Fs10706, and Fs13617 in *N. benthamiana* are due to immune suppression created by the iron-deficient environment generated by these effector proteins or through interactions with other host proteins, which still require further investigation.

Since the initial discovery of CFEM-like proteins in *Coccidioides immitis*, the distribution of CFEM proteins within the fungal community has significantly broadened. A notable pattern has emerged, indicating a clear differentiation in virulence among these CFEM members, which is associated with specific amino acid residues within the CFEM domain. However, both the sites and mechanisms of variation differ among various fungal species. For instance, in *C. graminicola*, only five of the ten CFEM effectors have been shown to possess the ability to suppress BAX-induced programmed cell death in *N. benthamiana* [29]. In contrast, among six CFEM effectors identified in *S. turcica*, only StCFEM12 was capable of inhibiting host cell death triggered by BAX [30]. Additionally, nine secreted small cysteine-rich proteins with CFEM domains have been identified in *V. dahliae*. Among these, two proteins, VdSCP76 and VdSCP77, are essential for the full virulence of *V. dahliae* on cotton and are capable of mediating the broad-spectrum suppression of immune responses. Furthermore, VdSCP76 and VdSCP77 suppress host immunity through a potential iron-binding site conserved in CFEM family members, and the Asn residue has been substituted with the Asp residue in VdSCP76 and VdSCP77 [6]. In this work, we identified three CFEM effectors (Fs08184, Fs10706, and Fs13617) from a total of nineteen CFEM domain-containing members in *F. sacchari*. These three effectors have been demonstrated to be essential for the full virulence of *F. sacchari* and are capable of mediating the broad-spectrum suppression of immune responses induced by typical effectors, suggesting that the CFEM members in *F. sacchari* differentially contribute to virulence. Further research has confirmed that the virulence differentiation among these FsCFEM members is closely related to two amino acid residues within the CFEM domain, as characterized by Asp and Phe residues in Fs08184, Fs10706, and Fs13617 (Figure 1). The C-ΔFs08184^D46NF56L^, C-ΔFs10706^D56NF66L^, and C-ΔFs13617^D49NF59L^ mutants failed to restore pathogenicity in sugarcane (Figure 5) and exhibit impaired growth in both iron-deficient and iron-rich conditions (Figure 6). Moreover, the overexpression of Fs08184^D46NF56L^, Fs10706^D56NF66L^, or Fs13617^D49NF59L^ did not induce the differential expression of genes associated with iron homeostasis in *N. benthamiana* (Figure 7), further supporting this conclusion. 

In summary, the findings presented herein provide essential foundational data for investigating the functional roles and virulence differentiation mechanisms of CFEM members in *F. sacchari* and will enhance our understanding of the interplay between iron homeostasis and host immune regulation at the interface of fungal–host interactions.

## 4. Materials and Methods

### 4.1. Growth of Plants and Microbes

The *Fusarium sacchari* was cultured on potato dextrose agar (PDA) or Czapek-DOX medium for 7 days at 28 °C. *Agrobacterium tumefaciens* AGL-1 and GV3101 were grown in Luria–Bertani (LB) medium supplemented with appropriate antibiotics at 28 °C for fungal transformation and transient expression experiments in *N. benthamiana*. Sugarcane cultivar GT42 was grown at 28 °C under a photoperiod with 14 h of light and 10 h of darkness until reaching the six-leaf stage for virulence assays [15]. *N. benthamiana* plants were maintained at 25 °C with a photoperiod of 16 h of light and 8 h of darkness for four weeks to facilitate transient expression experiments.

### 4.2. Transient Expression in Nicotiana benthamiana

To investigate the roles of two potential iron-binding sites that are conserved among CFEM members of *F. sacchari*, which mediate broad-spectrum suppression of cell death, the site-directed mutagenesis of amino acid residues critical to the function of *Fs08184* (*Fs08184^D46N^*, *Fs08184^F56L^*, and *Fs08184^D46NF56L^*), *Fs10706* (*Fs10706^D55N,^ Fs10706^F66L^*, and *Fs10706^D55NF65L^*), and *Fs13617* (*Fs13617^D49N^*, *Fs13617^F59L^*, and *Fs13617^D49NF59L^*) were amplified using overlapping PCR strategy. The plasmids previously constructed served as templates for this amplification process [16]. All sequences were individually cloned into the PVX vector pGR107 with a 3 × HA tag at C-terminal, using the CloneExpress Ultra One Step Cloning Kit (Vazyme). The constructs were then transformed into *A. tumefaciens* GV3101. The transient expression in *N. benthamiana* leaves was performed as previously described [28]. To investigate the suppression of cell death in *N. benthamiana*, the Bcl-2-associated X protein (BAX-PVX), Fs13311-PVX, Fs00548-PVX, and Fs05897-PVX constructs were injected at the indicated sites on the following day. The lesion areas were recorded at 3 to 5 days post inoculation, and are expressed as the ratio of the lesion area to the infiltrated area. Primers used in this study are listed in Supplementary Table S1.

### 4.3. Total Protein Extracts and Immunoprecipitation

To verify protein production during transient expression in *N. benthamiana*, tissue for protein extraction was collected 48–60 h post inoculation. Total protein was extracted using RIPA lysis buffer (89901, Thermo Fisher) and purified with Pierce Anti-HA magnetic beads (88837Thermo Scientific) following the manufacturer’s instructions. The proteins were subjected to 12% SDS-PAGE gel, followed by their transfer to a PVDF membrane, and subsequently immunoblotted with anti-HA antibody (AE008, ABclonal, Wuhan, China) and visualized using the Enhanced HRP-DAB Chromogenic Substrate Kit (Tiangen, Chian, PA110).

### 4.4. DAB Staining

Reactive oxygen species (ROS) staining in *N. benthamiana* leaves was detected using diaminobenzidine (DAB) solution, as described previously [31]. Plant tissues were infiltrated with DAB solution and incubated in the dark at room temperature for 24 h. Subsequently, they were decolorized with anhydrous ethanol before being photographed and recorded.

### 4.5. Gene Expression Analysis

Total plant RNA was extracted using the Eastep® Super Total RNA Extraction Kit (LS1040, Shanghai, Promega) following the manufacturer’s instructions. Complementary DNA (cDNA) was synthesized from 2 μg total RNA using the HiScript II Reverse Transcriptase Kit (R201-01, Vazyme, Nanjing, China) according to the manufacturer’s instructions. The cDNA was utilized as template for quantitative real-time PCR (qRT-PCR) using the ChamQ Universal SYBR qPCR Master Mix (Q711-02, Vazyme, Nanjing, China) with specific primer pairs, and all the reactions were performed on a LightCycler™ 96 Real-Time PCR System (Roche, Switzerland). *NbEFα* was employed as endogenous reference for gene expression analysis in *N. benthamiana*. All RT-qPCR experiments were conducted in triplicate biological replicates, each containing three technical replicates, and the relative transcript levels of specific genes to endogenous reference gene (*NbEFα*) were calculated using the 2^−ΔΔCT^ method [31]. Primers used in this study are listed in Supplementary Table S1.

### 4.6. Fungal Transformation

The *F. sacchari* knockout and complementary mutants (ΔFs08184, C-ΔFs08184, ΔFs10706, ΔFs13617, and C-ΔFs13617) used in this experiment were obtained from our previously study [16]. To generate complementation transformants, the sequences including the native promoter region, gene sequence (the wild-type *Fs10706*, as well as site-directed mutagenized genes *Fs08184^D46NF56L^*, *Fs10706^D55NF65L^*, and *Fs13617^D49NF59L^*), and native terminator region of targeted gene were amplified with corresponding primer pairs using overlapping PCR strategy. All sequences were individually introduced into the vector pDHtsk-G418, and the positive constructs were transformed into *A. tumefaciens* AGL1 for the fungal transformation as described previously [32]. The positive transformants were screened, isolated on potato dextrose agar medium supplemented with 200 mg/L Cefotaxime and 20 mg/mL G418, and subsequently verified through PCR using the appropriate primer pairs. Primers used in this study are listed in Supplementary Table S1.

### 4.7. Virulence Assays

The virulence assays for the knockout mutants and complementary mutants of *Fs08184*, *Fs10706*, and *Fs13617* were performed on sugarcane leaves from the GT42 cultivar, following previously established protocols [16]. All the *F. sacchari* strains were pre-cultured on the PDA medium for 7 days, and five-millimeter-wide colonized agar plugs were inoculated on the leaves from the six-leaf-stage seedling of GT42. The necrotic areas of the inoculated leaves were measured at 5 days post inoculation.

### 4.8. Mycelia Growth Assays

A total of 10 μL of 10^5^ conidia/mL *F. sacchari* conidia from wild-type CNO-1, knockout mutants, and complementary mutants of Fs08184, Fs10706, and Fs13617 were inoculated on Czapek-DOX medium containing different concentrations of Fe^2+^ and Fe^3+^ (-Fe, 0 mM EDTA-Fe^2+^ or EDTA-Fe^3+^; 1 × Fe^2+^, 35 mM EDTA-FeSO_4_; 1 × Fe^3+^, 35 mM EDTA-FeCl_3_; 2 × Fe^2+^, 70 mM EDTA-FeSO_4_; and 2 × Fe^3+^, 70 mM EDTA-FeCl_3_). The colony diameters were measured and statistically analyzed at 3 to 5 days post inoculation.

### 4.9. Statistical Analysis

The standard errors in all figures were calculated for each treatment with at least three replicates. Statistical significance analyses were performed using one-way ANOVA test in GraphPad Prism software (version 9.0). The different letters indicate significant difference at *p* < 0.05.

### 4.10. Accession Numbers

Sequence data from this article can be found in the GenBank databases under the following accession numbers: NbPR1, OQ675540.1; NbPR2, XM_019376146.1; NbHSR203J, NW_015819496.1; NbLOX, KC585517.1; NbHIN1 (KU195817); NbEF1, PQ008965.1; NbDTX43, XM_019390318.1; NbYSL1, XM_019391300.1; NbNRAMP2, OP972862.1; and NbNRAMP3, XM_009798480.1.

## Data Availability

All the data that support the findings of this study are available in the paper and Supplementary Materials.

## Supplementary Materials

The following supporting information can be downloaded at: https://www.mdpi.com/article/10.3390/ijms252312805/s1.
